# CRISPR-Cas9 mediated efficient PD-1 disruption on human primary T cells from cancer patients

**DOI:** 10.1038/srep20070

**Published:** 2016-01-28

**Authors:** Shu Su, Bian Hu, Jie Shao, Bin Shen, Juan Du, Yinan Du, Jiankui Zhou, Lixia Yu, Lianru Zhang, Fangjun Chen, Huizi Sha, Lei Cheng, Fanyan Meng, Zhengyun Zou, Xingxu Huang, Baorui Liu

**Affiliations:** 1 The Comprehensive Cancer Centre of Drum Tower Hospital, Medical School of Nanjing University & Clinical Cancer Institute of Nanjing University, Nanjing 210008, China; 2 MOE Key Laboratory of Model Animal for Disease Study, Model Animal Research Center of Nanjing University, National Resource Center for Mutant Mice, Nanjing 210061, China; 3 State Key Laboratory of Reproductive Medicine, Department of Histology and Embryology, Nanjing Medical University, Nanjing 210029, China; 4 School of Life Science and Technology, ShanghaiTech University, 100 Haike Rd., Pudong New Area, Shanghai 201210, China

## Abstract

Strategies that enhance the function of T cells are critical for immunotherapy. One negative regulator of T-cell activity is ligand PD-L1, which is expressed on dentritic cells (DCs) or some tumor cells, and functions through binding of programmed death-1 (PD-1) receptor on activated T cells. Here we described for the first time a non-viral mediated approach to reprogram primary human T cells by disruption of PD-1. We showed that the gene knockout of PD-1 by electroporation of plasmids encoding sgRNA and Cas9 was technically feasible. The disruption of inhibitory checkpoint gene PD-1 resulted in significant reduction of PD-1 expression but didn’t affect the viability of primary human T cells during the prolonged *in vitro* culture. Cellular immune response of the gene modified T cells was characterized by up-regulated IFN-γ production and enhanced cytotoxicity. These results suggest that we have demonstrated an approach for efficient checkpoint inhibitor disruption in T cells, providing a new strategy for targeting checkpoint inhibitors, which could potentialy be useful to improve the efficacy of T-cell based adoptive therapies.

Checkpoint blocking are revolutionizing treatment options and expectations for cancer patients. Instead of directly acting on the tumor to induce tumor cell death, checkpoint inhibitors enhance or stimulate antitumor immune responses to eliminate cancer cells^1^. As it has been proved that the observed immune responses by the immunization of tumor vaccines does not always confer clinical benefit^2^,^3^,^4^. The majority of vaccine-induced T cells have low recognition efficiency and the lytic ability on tumor cells, and this may account for their lack of clinical effect^5^,^6^,^7^. Monoclonal antibodies (mAbs) blocking immune checkpoint receptors have recently emerged as promising therapeutics to overcome the above shortcomings. PD-1 is present on activated T cells and regulatory T (Treg) cells, and its ligand PD-L1 is expressed by most cell types including tumor cells and DCs^8^,^9^,^10^. Anti-PD-1 antibody produced objective responses in approximately one in four to one in five patients with non-small-cell lung cancer, melanoma, or renal-cell cancer^11^. However, it has to be noted that most tissues rely on PD-L1 expression to limit T-cell response, so the systemic administration of PD-L1/PD-1 blocking antibodies still carries the risk of breaking peripheral tolerance^12^.

In the more frontier point of view, cell-intrinsic disruption of immune checkpoints by gene targeting in T-cells is likely to display a better safety profile than the systemic administration of blocking antibody^13^. To this end, the delivery of genome editing agents to T-cells is a crucial aspect of their successful application to adoptive cell transfer therapy (ACT). The first generation gene disruption technique zinc finger nucleases (ZFNs) has already entered clinical development, but the complicated design and labor-intensive cost limit its wide spread in non-specialized laboratories and may slow down its clinical developments^14^. The generation of arrays of TALEN (Transcription activator-like effector nucleases), another kind of gene targeting approach, is incompatible with efficient reverse transcription and required for the delivery of genetic agents using lentivirus^15^, which may be also labor-intensive and thereby limiting its use in the clinic.

Recently described RNA-guided endonucleases, CRISPR (clustered regularly interspaced short palindromic repeats) and CRISPR-associated (Cas) 9, provide an attractive alternative to genome editing compared with protein-guided nucleases. The CRISPR-Cas9 system conferring targeted gene editing by small RNAs that guide the Cas9 nuclease to the target site through base pairing^16^ has been demonstrated as an easy-handle, highly specific, efficient approach for engineering eukaryotic genomes^17^. In our previous work we had achieved efficient gene targeting in mice and rats by co-injection of one-cell-stage embryos with Cas9 mRNA and sgRNAs^18^,^19^,^20^ and had recently demonstrated success in Cynomolgus monkeys^21^. CRISPR-Cas9-mediated genome editing is considered to be the holy grail of genome editing, but current lack of evidence of this technology on the use of human primary T cells may limit its use on humans and hinder its way towards a clinical platform^13^.

Given the beneficial effects of PD-1 blockade in improving the quality of antitumor effect of tumor reactive T cells in varies cancer types, here we set out to explore the cell intrinsic disruption of this immune checkpoint by CRISPR-Cas9 genome editing and to find a non-viral mediated transfection method which is in favor of clinical application. Herein, we show that the gene knockout of PD-1 by electroporation of plasmids encoding sgRNA-Cas9 DNA is technically feasible and efficient, and this genome disruption in primary human lymphocytes sustained over prolonged *in vitro* culture in the presence of antigen stimulation. Moreover, this electroporation mediated intrinsic PD-1 gene disruption will not affect the proliferation capacity of primary T cells whereas enhance cellular immune responses and cytotoxicity on tumor cell lines. These results underscore the therapeutic potential of a non-viral mediated CRISPR-Cas9 genome editing method for the disruption of immune inhibitory checkpoints, and may realize its clinical application in adoptive T-cell transfer therapy of cancer.

## Results

### The design and validation of sgRNA targeting hPD-1

**Figure 1:**
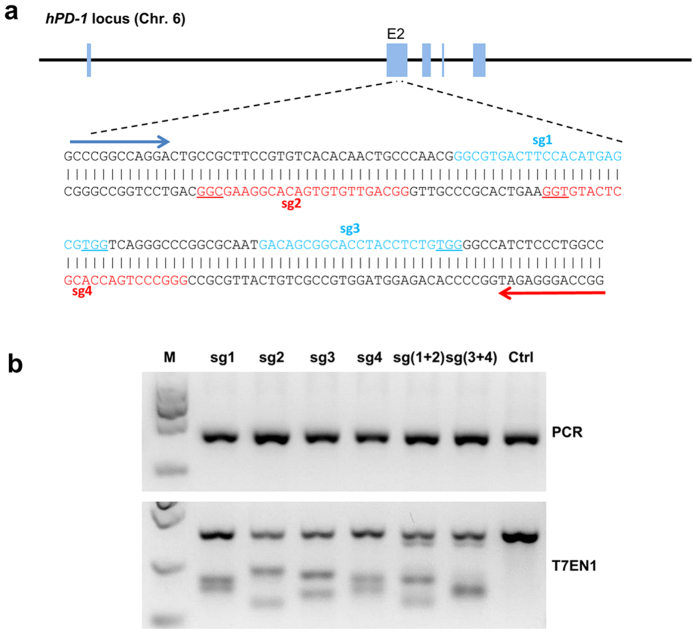
Evaluation of hPD-1 sgRNA:Cas9-mediated modifications of human PD-1. (**a**) Schematic diagram of sgRNAs targeting at hPD-1 Exon 2 locus. The sgRNAs targeting sites on the sense strand are colored with blue while those on the antisense are colored with red. PAM sequences are underlined. (**b**) Detection of sgRNA:Cas9-mediated cleavage of hPD-1 by PCR and T7EN1 cleavage assay. M, DNA marker. sg1, sgRNA 1; sg2, sgRNA 2; sg3, sgRNA 3; sg4, sgRNA 4; sg (1 + 2), sgRNA 1 combined with sgRNA 2; sg (3 + 4), sgRNA 3 combined with sgRNA 4. Con, negative control. The above experiments have been repeated 3 times with similar results.

Previous works have shown that simultaneous use of dual sgRNAs to target an individual gene significantly improved the Cas9-mediated genome targeting efficiency and reduce the site-dependent off-target effect^22^. So we selected 2 pairs of targeted sites on exon 2 (Fig. 1a). All sgRNA expression vectors were co-transfected into HeLa cells separately with Cas9 expression vector to test their efficiency. Blasticidin (5 μg/ml) and puromycin (1 μg/ml) were added 24 h after transfection for positive selection. Genomic DNA was isolated from cells harvested 72 h after transfection and screened for the presence of site-specific gene modification by PCR amplification of regions surrounding the target sites as well as T7EN1 cleavage assay. The cleavage bands were all clearly observed (Fig. 1b) and various indels were detected by the sequencing (Supplemental Fig. 1). Mutation sizes ranged from −86 to +51 at the efficiency of 61.9% for sg1, 52.6% for sg2, 40% for sg3, 52.6% for sg4, 47.6% for sg (1 + 2) and 38.9% for sg (3 + 4), respectively. These data demonstrated that the selected sgRNAs worked effectively with Cas9 on human genomes. We chose sg (1 + 2) in our following experiments.

### Optimizing Cas9/sgRNAs delivery of primary T cells

**Figure 2:**
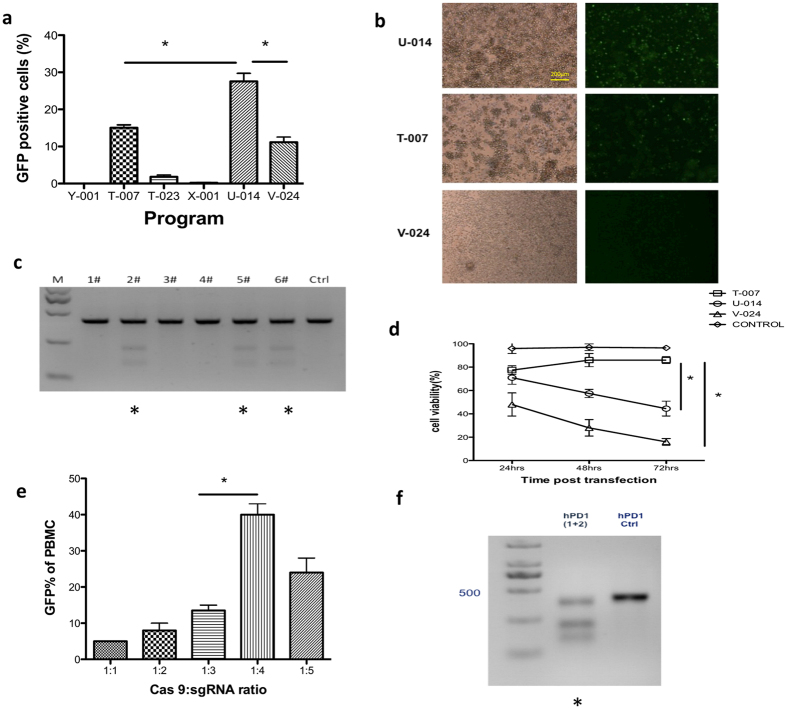
Optimized conditions for Cas9 and sgRNA hPD-1 plasmids co-transfection of human primary T cells. PBMC from healthy donors were co-transfected with pST1374-Cas9-GFP and pGL3-U6-hPD-1-sgRNA and the expression of GFP was observed 24 h after electroporation. (**a**) Different nucleofection programs and the percentage of GFP positive cells assessed by flow cytometry. (**b**) Representative images of the transfected cell by light microscope and fluorescence microscope. (**c**) Detection of sgRNA1:Cas9-mediated cleavage of hPD-1 by T7EN1 cleavage assay, #1: Y-001; #2: T-007; #3: T-023; #4: X-001; #5: U-014; #6: V-024. Samples with cleavage bands were marked with *. (**d**) Cell viability 24, 48 and 72 h after transfection by Trypan blue exclusion assay. (**e**) The transfection efficacy of different molar ratio of pST1374-Cas9-GFP and pGL3-U6-hPD-1-sgRNA evaluated by GFP expression. (**f**) Detection of sgRNA1:Cas9-mediated cleavage of hPD-1 by T7EN1 cleavage assay using program T-007 with a molar ratio of 1:4 (pST1374-Cas9-GFP:pGL3-U6-hPD-1-sgRNA). Data shown are mean ± SD of 3 independent experiments. p < 0.05.

We next aimed at optimizing conditions for the co-transfection of Cas9/sgRNA expression plasmids on primary T cells via electroporation. For this purpose, we used reporter plasmids pST1374-Cas9-GFP encoding green fluorescent protein (GFP) tagged Cas9 to evaluate the transfection efficacy through the co-electroporation with pST1374-Cas9-GFP and pGL3-U6-hPD-1-sgRNA (1 + 2). Here we referred to several related research based on primary T cells transfection by the Nucleofecter platform and expanded our experimental program beyond the Lonza protocol. To this end, a mixture of 5 μg of pST1374-Cas9-GFP and 10 μg of pGL3-U6-hPD-1-sgRNA plasmids was used, and different programs (Y-001, T-007, T-023, X-001, U-014, V-024) were applied. GFP expression was determined 24 h later by fluorescence microscope or flow cytometry. We found that higher transfection efficacy was obtained with program U-014 than with V-024 and T-007 (Fig. 2a,b, p = 0.0231 and p = 0.0316). The T7EN1 cleavage assay confirmed the PD-1 mutation of T cells (Fig. 2c). Notably, during the following days, significant improved cell viability was observed with program T-007 than with U-014 and V-024 shown by Trypan blue exclusion assay (Fig. 2d, p = 0.0353 and p = 0.0058). Therefore, T-007 was used as the optimal program for the following experiments. In addition, we found significant increase of GFP expression when plasmids were delivered at a molar ratio of 1:4 (pST1374-Cas9-GFP:pGL3-U6-hPD-1-sgRNA) (Fig. 2e). To determine the mutation of PD-1, T7EN1 cleavage assay was applied and the gene knockout efficacy was proved (Fig. 2f). These results indicated that human PBMC-derived T cells were efficiently modified by sgRNA:Cas 9 system through the electroporation of plasmids.

### Cas9-mediated efficient PD-1 KO in primary T cells of patients

**Figure 3:**
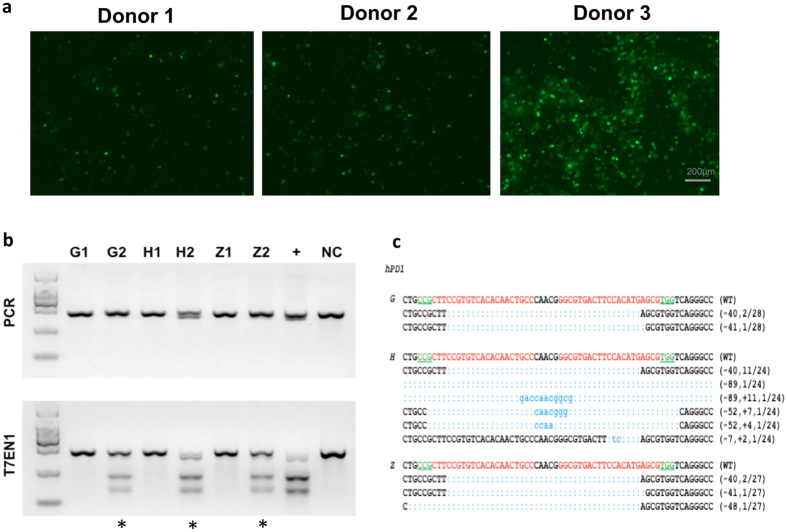
Cas9 mediated efficient hPD-1 KO in primary human T cells of healthy donors and patients. Freshly isolated PBMC were activated *in vitro* by IFN-γ for 3 d and IL-2 and aCD3 for 2 d and were transfected with pST1374-Cas9-GFP and pGL3-U6-hPD-1-sgRNA plasmids for each reaction. Sample G and Z stand for two individual patients and H stands for a healthy donor. (**a**) The GFP expression was evaluated by fluorescence microscope 24 h after electroporation (Donor 1 and Donor 2 are patients. Donor 3 is a representative of healthy donor). (**b**) PCR products were amplified and subjected to T7EN1 cleavage assay. Samples with cleavage bands were marked with an asterisk “*”. Sample G1/H1/Z1 represent control T cells and G2/H2/Z2 represent hPD-1 KO T cells. “**+**” represents for the positive control with the cleavage bands detected on HeLa cell line. NC, negative control. (**c**) DNA sequences of marked samples. TA clones from the PCR products were analyzed by DNA sequencing. The PAM sequences are underlined and highlighted in green; the targeting sequences in red; the mutations in blue, lower case; deletions (−), and insertions (+). The above experiments have been repeated 3 times with similar results.

To test whether the Cas9-mediated gene knockout was as efficient in patients as in healthy donors, primary T cells from two late stage cancer patients and healthy donors were transfected. Freshly isolated PBMC were activated for 3 d. A total of 10 μg of pST1374-Cas9-GFP and 20 μg of pGL3-U6-hPD-1-sgRNA plasmids were used for each reaction. The GFP expression was evaluated by fluorescence microscope 24 h after electroporation. We found that PBMC-derived T cells from patients (Donor 1 and Donor 2) (Fig. 3a) achieved less transfection efficacy comparing with T cells from healthy donor (Donor 3) (Fig. 3a). Over 85% viable cells were observed in all three donors evaluated by Trypan blue exclusion assay (Table 1). To determine the subsets of T cells, surface expression of CD3, CD4 and CD8 were calculated on day 7 of culture. The results showed that over 85% of cells were CD3^+^ T cells and over 80% of CD3^+^ T cells were CD8^+^ cells in all three donors (Table 1). PCR results showed there harbored large fragment deletion on samples from healthy donor (H2), also indicating the higher efficacy of gene disruption than the other two donors (G2 and Z2). The subsequent T7EN1 assay and sequencing further confirmed that the mutation of PD-1 was successfully achieved in all three donors (Fig. 3b). Further characterization of the cleavage by sequencing showed, different indels were detected in all the donors with various mutation sizes, the efficacy varied among donors (10.71% in Donor 1, 14.81% in Donor 2, 66.67% in Donor 3) (Fig. 3c). These results demonstrated that the efficient disruption of gene PD-1 is achieved by sgRNA:Cas9 system although the efficacy of the gene modifications varied among different individuals.

### The proliferation of gene edited primary T cells and the sustained knockout of PD-1 during the prolonged culture conditions

**Figure 4:**
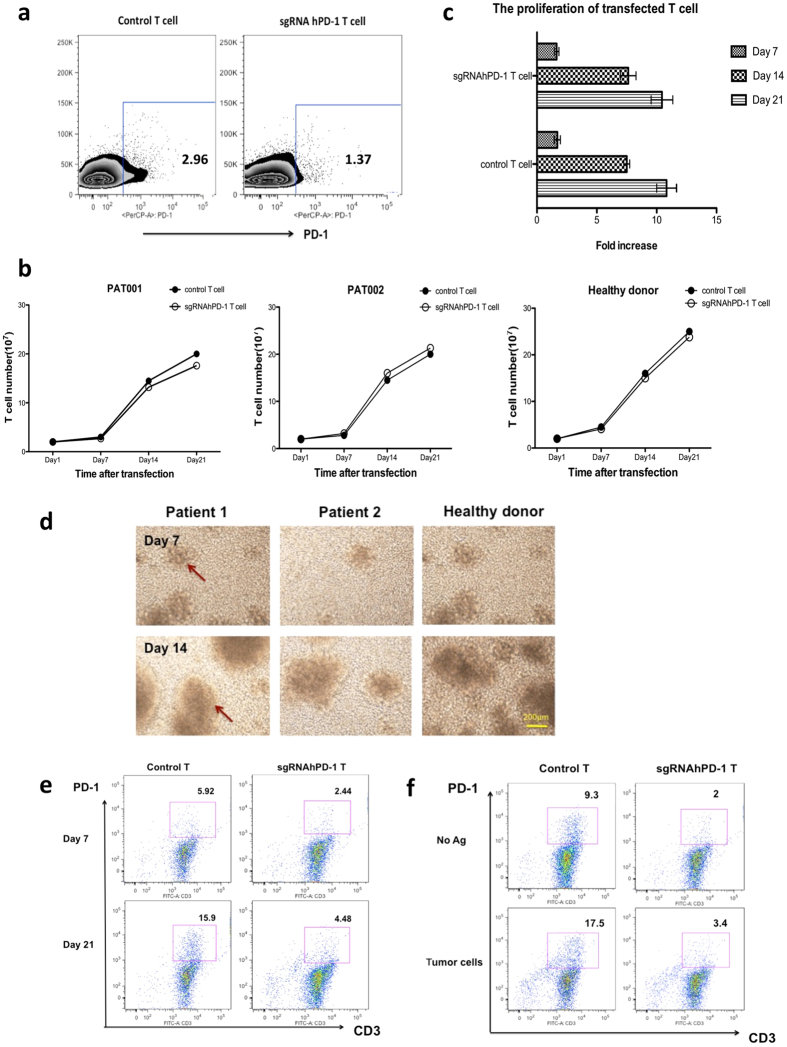
The analysis of proliferation of gene modified primary T cells and the sustained knockout of hPD-1 during the prolonged culture conditions. (**a**) PD-1 expression on CD3^+^ T cells was determined by flow cytometry 48 h post transfection. (**b,c**) Control T cells and sgRNA:Cas9-treated T cells were cultured *in vitro* upon stimulation with IL-2 for 21 d. The total cell numbers were counted every 7 d and the fold increase on day 7, day 14 and day 21 were evaluated. (**d**) T cell clones were observed around day 7, grew largely and increased during the following cultured days shown by light microscope. (**e,f**) The expression of hPD-1 on CD3^+^ T cells was determined by flow cytometry 7 and 21 d post transfection stimulated by peptide-pulsed autologous DCs and irradiated tumor cells, respectively. We depicted a representative out of three experiments yielding similar results. Data shown are mean ± SD of 3 independent experiments and we depicted a representative out of three experiments yielding similar results.

As adoptive T cell therapies require relatively long culture of T cells *in vitro*, we are curious how long the down regulation of PD-1 lasted by our gene disruption method. To this end, we first assessed the surface expression of PD-1 48 h post transfection. Here we depicted a representative out of three experiments yielding similar results. The percentage of PD-1^+^ T cells was 2.96% on control T cells and was 1.37% on the sgRNA:Cas9 modified T cells (Fig. 4a), though the baseline expression of PD-1 was relatively low. Then, we assessed the capacity of sgRNA:Cas9-treated T cells to proliferate *in vitro* upon stimulation with IL-2 by counting the total cell numbers. Over a period of 21 d, we found the proliferation of primary T cells of all these donors was not significantly affected by the disruption of PD-1 during the prolonged culture period, ranging from 2 × 10^7 ^cells on day 7 to 20 × 10^7^ on day 21, and the fold increase was 1.68 ± 0.03, 7.57 ± 0.09 and 10.64 ± 0.27 on day 7 (p = 0.8816), day 14 (p = 0.8557) and day 21 (p = 0.7705), respectively (Fig. 4b,c). T cell clones were observed around day 7 and grew largely during the following days, indicating good proliferation and activation of the T cells (Fig. 4d). Meanwhile, T cells from a healthy donor were transfected and stimulated by autologous DCs loaded with immunogenic peptides for another two weeks. The surface expression of PD-1 of CD3^+^ T cells increased from 2.44% at the baseline to 4.48% of the PD-1 KO group while it increased from 5.92% to 15.9% in control group (Fig. 4e). To further mimic the *in vivo* environment when T cells encounter with whole tumor antigen, we co-cultured the gene modified T cells with irradiated PD-L1-high tumor cells. We observed that only 3.4% of sgRNA:Cas9 modified T cells express PD-1 while 17.5% of control T cells express PD-1 by the stimulation of whole tumor antigen (Fig. 4f). Taken together, these data represented that the proliferation capability was not affected by the disruption of PD-1 on primary T cells and that the efficient disruption of PD-1 sustained during the prolonged culture with the stimulation of tumor antigens.

### The characterization of the cultured T cells by sgRNA:Cas9 mediated knock out of PD-1

**Figure 5:**
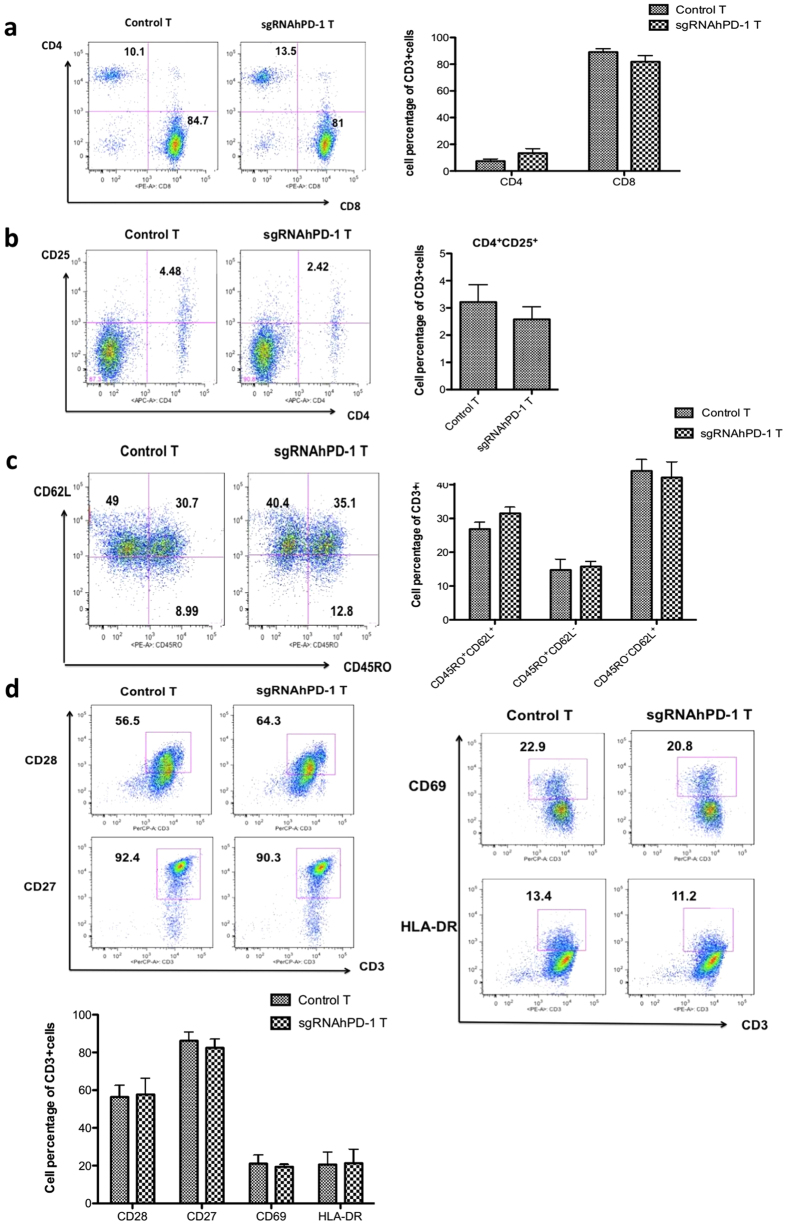
The phenotype of the *in vitro* cultured T cell by sgRNA:Cas9 mediated knock out of PD-1. T cells after electroporation on day 21 were harvested to evaluate the phenotype change of cultured T cells after gene editing from patients and healthy donors. (**a**) CD4^+^ and CD8^+^ cells were analyzed by gating on CD3^+^ cells (**b**) CD4^+^CD25^+^ cells were analyzed by gating on CD3^+^ cells. (**c**) CD45RO^+^CD62L^+^, CD45RO^+^CD62L^−^ and CD45RO^−^CD62L^+^ cells were analyzed by gating on CD3^+^ cells. (**d**) Cell surface expression of the activation marker CD28, CD27, CD69 and HLA-DR were measured on CD3^+^ cells. Graphs show quantification of FACs data. Data shown are mean ± SD of 3 independent experiments.

To characterize the cultured T cells with PD-1 disruption, we harvested T cells on day 21 post transfection. The T cell subsets were determined by the expression of CD4, CD8 and characterized with memory or activation markers, including CD28, CD27, CD69 and HLA-DR. The results showed, the PD-1 knock out T cells did not exhibit any significant change on the expression of CD4 (7.43 ± 2.78% on control T cells vs 13.36 ± 6% on sgRNAhPD-1 T cells, p = 0.1957) or CD8 (89.10 ± 4.5% on control T cells vs 81.87 ± 7.82% on sgRNAhPD-1 T cells, p = 0.2367) during the extended *in vitro* culture after gene editing (Fig. 5a). Although the decrease of CD4^+^ CD25^+^ cells on PD-1 KO T cells (4.48% vs. 2.42%) was observed in one of the donor (Fig. 5b), there was no statistical significance, p = 0.4604. It is noteworthy that, we did not find any significant change of the memory markers (Fig. 5c), including central memory CD45RO^+^CD62L^+^ T cell (26.87 ± 3.48% vs 31.53 ± 3.1%, p = 0.1583), effector memory CD45RO^+^CD62L^−^ T cell (14.76 ± 5.44% vs 15.77 ± 2.58%, p = 0.7858) and naïve T cell (44.1 ± 5.79% vs 42.1 ± 8.08%, p = 0.7451). No difference of the activation marker CD28, CD27, CD69 and HLA-DR on CD3^+^ T cells was detected between the sgRNAhPD-1 T cells and control T cells (p > 0.01) (Fig. 5d). These results indicated that the CD4 or CD8 subsets constitution or the memory and activation status of the T cells is stable with sgRNA:Cas9-mediated PD-1 disruption.

### Enhanced cytokine secretion by the disruption of PD-1 in primary T cells of healthy donors and patients

**Figure 6:**
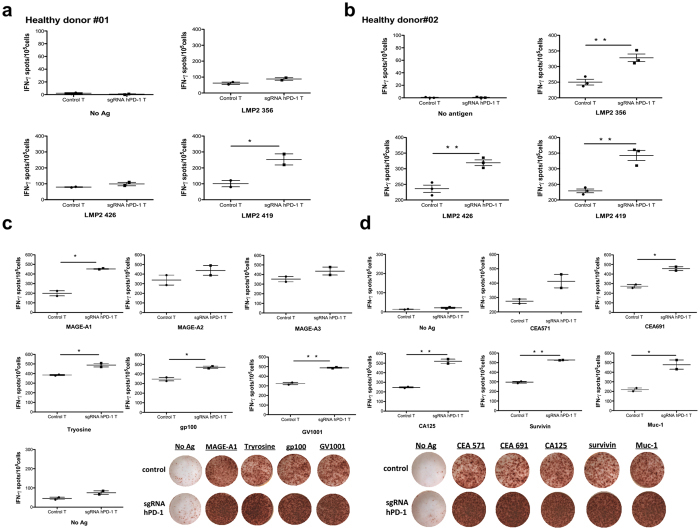
The enhanced cytokine production by the Cas9-mediated hPD-1 KO in primary T cells. sgRNA hPD-1:Cas9 modified primary T cells and control T cells from healthy donors or patients were cultured in IL-2 after electroporation for 7 d and stimulated by peptides pulsed autologous DCs for 20 h. The IFN- γ secreting cells was evaluated by Elispot. (**a,b**) T cells from two EBV serum positive healthy donors were stimulated by LMP2a peptides pulsed autologous DCs. (**c**) T cells from a melanoma patient was stimulated by melanoma associated peptides pulsed autologous DCs. (**d**) T cells from a gastric cancer patients were stimulated by gastric cancer associated peptides pulsed autologous DCs. All values shown are mean ± SD of triplicate or duplicate measurements and have been repeated 3 times with similar results. *p < 0.05; **p < 0.005.

Next, we assessed effector capabilities of reprogrammed primary T cells upon antigen stimulation using interferon-γ Elispot assays. sgRNA hPD-1:Cas9 modified primary T cells and control T cells from healthy donors were cultured in IL-2 for 7 d after electroporation and stimulated by autologous DCs loaded with LMP2a peptides for 20 h. Interferon-γ secretion was significantly higher on PD-1 KO T cells by the stimulation of HLA-A24 restricted LMP2a 419 or HLA-A02 restricted epitope LMP2a 356 and LMP2a 426 comparing with control T cells of HLA-matched donors, respectively (HD#01: LMP2a 356, p = 0.0629; LMP2a 426, p = 0.1102, LMP2a 419, p = 0.0316) (Fig. 6a); (HD#02: LMP2a356, p = 0.0037; LMP2a426, p = 0.0026; LMP2a419, p = 0.0014) (Fig. 6b). Similarly, we evaluated the immune response of sgRNA hPD-1:Cas9 modified primary T cells from two late stage cancer patients by stimulating their T cells with the tumor associated HLA-epitope matched peptides. It also exhibited a significant enhancement of IFN-γ production of the PD-1 KO groups (melanoma patient: MAGE-A1, p = 0.0053; Tyrosine, p = 0.0163; gp100, p = 0.0149; GV1001, p = 0.0022; MAGE-A2, p = 0.1499; MAGE-A3, p = 0.1168) (Fig. 6c); (gastric cancer patient : CEA571, p = 0.0519; CEA691, p = 0.0209; CA125, p = 0.0035; Survivin, p = 0.0008; Muc-1, p = 0.0176) (Fig. 6d). In addition, we selected 3 more cancer patients who had pre-existed immune responses to the personalized peptide and stimulated modified T cells from these patients with four reactive peptides antigens in the presence of their autologous DCs. In two of these patients, significant enhancement of IFN-γ production of the sgRNAhPD-1 T cells were observed (patient 03: Her-2, p = 0.0094; CA125, p = 0.0088; hTERT, p = 0.0058; PTH-rp, p = 0.0147) (Supplementary Figure 2a) (patient 05: URLCC, p = 0.045; EGFR, p = 0.0038; EZHZ-291, p = 0.0655; EZHZ-735, p = 0.0014) (Supplementary Figure 2b). The left one without functional enhancement may most possibly due to the low transfect efficacy of the donor’s primary T cells (data not shown). Together, these data provided proof of the successful and efficient reprogramming of primary T cells applying sgRNA:Cas9 system and indicated that the disruption of PD-1 enhanced cellular immune response towards antigen stimulation in a HLA dependent manner.

### Enhanced cytotoxicity by the disruption of PD-1 in primary T cells

**Figure 7:**
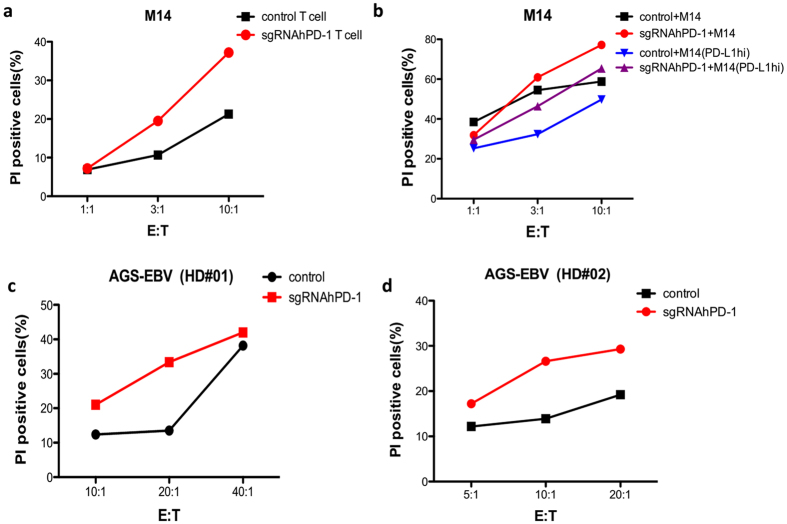
Enhanced cytotoxicity of the hPD-1 KO primary T cells. Patient or healthy donor derived T cells reprogrammed by sgRNA:Cas9 or control were cultured *in vitro* with IL-2 for 7 ~ 10 d and co-cultured with PD-L1 expressing tumor cells in different effector to target cell ratio. The cytotoxic reactivity of the effector T cells was measured using CFSE/PI cytotoxicity assay. The relative percentage of double-positive cells out of the CFSE-labeled population (tumor cells) is shown. (**a**) The hPD-1 KO T cells or control T cells from a melanoma patient were co-cultured with CFSE labeled M14 cells at ratio (E:T) of 1:1, 3:1, 10:1, respectively. After 6 h, PI was added and the cells were analyzed by flow cytometry. (**b**) The hPD-1 KO T cell or control T cells from a melanoma patient were co-cultured with CFSE labeled PD-L1-lo-M14 or PD-L1-hi-M14 cells at ratio (E:T) of 1:1, 3:1, 10:1, respectively. After 6 h, PI was added and the cells were analyzed by flow cytometry. (**c,d**) The hPD-1 KO T cells or control T cells from healthy donor #01 and healthy donor #02 were co-cultured with CFSE labeled AGS-EBV cells at ratio (E:T) of 5:1, 10:1, 20:1, or 10:1, 20:1, 40:1, respectively. After 16 h, PI was added and the cells were analyzed by flow cytometry. The above experiments have been repeated 3 times with similar results.

As we demonstrated above, the cellular immune response of T cells was enhanced by the sgRNA:Cas9-mediated KO of PD-1. We subsequently sought to determine if a similar enhancing effect would be observed on the lytic activity of tumor cells *in vitro*. Thus, we co-incubated melanoma patient-derived transduced T cells with PD-L1 expressing M14 melanoma cells. The cytotoxic reactivity of the effector T cells was determined by CFSE/PI cytotoxicity assay. The PD-1 KO T cells from this patient showed enhanced lytic capability on M14 cells in a dose dependent manner (Fig. 7a, red line vs. black line). Further, we demonstrated in accordance with the previous reports that when M14 tumor cells were cultured in the condition of IFN-γ and TNF-α, PD-L1 expression were up-regulated. We subsequently evaluate the cytotoxicity of patient derived T cell on PD-L1 high M14 cells and found decreased tumor lysis comparing with its PD-L1 low counter-part (Fig. 7b, blue line vs. black line). Moreover, this suppression of cytotoxicity was overcome by replacing the control T cells with the PD-1 KO T cells from the same donor (Fig. 7b, purple line vs. blue line). In addition, we observed an increase of lytic ability of PD-1 KO T cells from two healthy donors on a PD-L1 expressing gastric cell line (Fig. 7c,d, red line vs. black line). Thus, the disruption of PD-1 by sgRNA:Cas9 system improved the cytotoxicity of T cells from patients or healthy donors on tumor cell lines.

## Discussion

The effective activation of the tumor reactive T cells and the suppression of checkpoint inhibitor has long been the key problem of immunotherapy. Recently, the utilization of checkpoint blockade targeting the PD-1/PD-L1 pathway has shown remarkable antitumor responses in patients with advanced melanoma, lung cancer and against other cancers with durable clinical responses^23^,^24^,^25^,^26^. T cells activated in the absence of PD-L1/PD-1 co-stimulation are functional activated, exhibiting increased proliferation by the stimulation of DCs or tumors and produce higher levels of Th-1 cytokines, in particular IFN-γ, IL-2 and TNF-α and enhanced lytic activities^27^. However, most of these studies have been performed using either blocking antibodies or RNA interference with siRNAs^28^,^29^, or by expression of the soluble extracellular part of PD-1 (sPD-1) or PD-L1 (sPD-L1) in human monocyte-derived DCs during antigen presentation^12^.

Adoptive cell therapy using autologous gene editing T cells such as TCR-T or CAR-T has emerged as promising approach for the treatment of cancers^30^. There is demonstration that blockade of the PD-1 immunosuppressive pathway using an anti-PD-1 antibody significantly enhance the anti-tumor efficacy of genetically modified T cells expressing a chimeric antigen receptor (CAR)^31^. Despite the dramatic benefit achieved by these strategies, it has to be noted that in one hand, sustained expansion of PD-1-expressing CTLs by the stimulation of tumor antigens *in vitro* and *in vivo* may require continuous treatment with anti-PD-1 antibody which is costly, on the other hand, the long term systematic administration of the blocking antibody carries the risk of breaking immune tolerance may cause immune attack of normal tissues. In addition, RNA interference with siRNAs on T cell may be temporary and less efficient.

In our study, we described, for the first time, a new approach of inhibiting PD-1/PD-L1 co-stimulation by directly disrupting genome PD-1 expression on human primary T cells through the Cas9:sgRNA gene knock out system. This was achieved by electro-co-transfer of two DNA plasmids into cultured human T cells. A major obstacle of editing primary T cell is the low transfection efficacy. Here, we made use of nucleofection to achieve higher transfection efficacy and better cell viability. This non-viral mediated gene disruption method has the advantage of clinical application at affordable costs^32^. To optimize the established protocol for co-transfection of primary T cell, by using a GFP reporting plasmid, we observed significantly improved cell viability with program T-007 than with U-014 and V-024 which are mostly used in the studies of T cell editing^33^. In addition, the ratio of co-transfection of two plasmid were vital for the efficient gene disruption of sgRNA:Cas 9 system.

Interestingly, our approach to disrupt PD-1 expression on human T cells was successfully utilized on several cancer patients and healthy donors as the requirement of T cell editing for adoptive transfer of patient’s autologous lymphocytes or in some cases allogenetic lymphocytes of healthy donors. We found that PBMC-derived T cells from patients achieved less transfection efficacy comparing with T cells from healthy donors. Those functional impaired T cells may account for the low sensitivity to electroporation. The phenomenon inspired us to testify this approach firstly in the allogenetic adoptive therapy of cancer treatment using PBMC from healthy donors. Moreover, we observed for over 3 w after PD-1 gene disruption, and found no significant differences in the cell expansion rate between sgRNA:Cas9-treated cells and control cells, which encouraged us to further apply this approach into clinical application in future. In addition, the activation induced up-regulation of PD-1 was disrupted through the *in vitro* expansion stimulated by peptides pulsed DCs or irradiated whole tumor cells. As we know that the most popular schedule for T cell *in vitro* culture would not surpass 3 ~ 4 w.

Previous studies already demonstrated that blockade of PD-1/PDL1 by mAb improved IFN-γ production as well as cytotoxicity both *in vitro* and *in vivo*^29^,^31^,^34^. Here we also demonstrated that these sgRNA hPD-1:Cas9 modified primary T cells from healthy donors or late stage cancer patients exhibited enhanced IFN-γ production by stimulating with the related peptide antigens and at the same time we found improved tumor cells lysis by the disruption of PD-1 which may due to the reversed immune resistance mediated by PD-1/PDL1 interaction. IFN-γ is one of Th1 cytokines which mediated cellular immune response and activate cytotoxic T cells, indirectly regulate tumors lysis by several mechanisms^35^. Therefore, we believe in our case, IFN-γ activates cytotoxicity indirectly. Additionally, in our system using Cas9:sgRNA mediated gene editing of T cells from both patients and healthy donors, improved cytotoxicity on tumor cell lines was clarified on two PD-L1 positive target cell lines and further testified by induction of PD-L1 expression on the target cell. The expression of its receptor PD-L1 should be taken into consideration for good outcome of utilizing PD-1/PD-L1 inhibiting strategy^36^.

Nevertheless, our study did not precisely focus on a specific antigen and our exploration was nor clearly clarified in an antigen dependent manner. We are currently explore PD-1 disruption of T cells by generating CTL targeting on specific tumor antigen, as previous studies reported that sustained expansion of PD-1-expressing CTLs may require continuous treatment to interrupt PD-1/PD-L1 co-stimulation^27^. In addition, as it is difficult to obtain large number of PD-1 high expression T cells from periphery lymphocytes, the difference of the functional analyzes between our “PD-1 KO cells” and “control cells” need further study. We sought to use the approach in the following research by changing the model into PD-1 high expression tumor infiltrating lymphocytes in solid tumors or tumor associated lymphocytes in ascites or pleural effusion of cancer patients, as they expose intensively to tumor antigens and acquired adaptive immune resistance that mediates resistance to immunotherapies^37^.

In conclusion, we established an sgRNA:Cas9-basd effective gene disruption method for the highly efficient disruption of PD-1 on primary human T cells. This easy handling technique has great potential to achieve nice effect. And this electroporation mediated approach provides an alternative to labor-intensive and time-consuming viral-mediated gene transfer methods. Therefore, our gene editing method might be suited for both research and clinical applications and we are confident that it will be beneficial to cancer adoptive cell transfer treatments using tumor-specific lymphocytes in the near future.

## Materials and Methods

### Ethics statement

All the experimental methods were carried out in accordance with the approved guidelines. The blood collection procedure was carried out in accordance with the guidelines verified and approved by the Ethics Committee of Drum Tower Hospital. All donors signed an informed consent for scientific research statement.

### Plasmid expression vectors

The Cas9 expression construct pST1374-Cas9-N-NLS-Flag-linker (Addgene 44758) was modified by adding an EGFP sequence to its C terminal. Oligos (Supplementary Table 1) for generation of sgRNA expression plasmids were annealed and cloned into the BsaI sites of pGL3-U6 sgRNA-PGK-Puro vector. The pGL3-dual U6-sgRNAs-PGK-Puro vector was produced based on pGL3-U6 sgRNA-PGK-Puro vector (Addgene 51133), which was modified by removal the small fragment between two BsaI sites and replaced with a ccdb suicide gene. The BsaI sites were also substituted for Esp3I sites. Taken the origin U6 promoter and sgRNA scaffold on pGL3-U6 sgRNA-PGK-Puro as the 1^st^ sgRNA promoter and the 2^nd^ sgRNA structure, primers containing pairs of 20 bp targeted sequences (Supplementary Table 2) were used to PCR-amplify the 1^st^ sgRNA scaffold and the 2^nd^ U6 promoter from a pUC57-sgRNA-U6 plasmid. The PCR products were then digested with Esp3I and the ccdb gene was replaced, forming the tandem dual sgRNA expression vectors.

### Preparation of primary human PBMCs

Apheresis specimens were collected from stage III/IV cancer patients or healthy donors. PBMCs were isolated by centrifugation on a Ficoll density gradient and suspended in AIM-V medium (Gibico, USA). Cells were frozen in 90% FBS serum (Gibico, USA), and 10% dimethyl sulfoxide (Sigma, USA). All PBMCs were used for experiments or stored in a secure liquid nitrogen freezer until use.

### T cell activation and electroporation

PBMC were cultured by adherence for 1 ~ 2 h and non-adherent cells were moved and suspended in AIM-V medium supplemented with 1000 U/ml IFN-γ on day 1 and 50ng/ml OKT-3 (eBioscienc, USA) and 300 U/ml of human recombinant IL-2 (eBioscienc, USA) on day 2 for 2 ~ 3 d. Cells were transfected with the intended plasmids by Nucleo-fector 2B (Lonza, Germany) using the Amaxa Human T cells Nucleofector Kit, VPA-1002 (Lonza, Germany). 5 ~ 10 × 10^6 ^cells were washed twice with DPBS by centrifuging at 800 rpm for 5 m and resuspended in 100 μl transfection buffer and then transferred into the electroporation cuvette. Program T-007 was selected for both high transfection and high efficiency. After electroporation, cells were resuspended in 500 μl pre-warmed AIM-V medium containing 10% FBS and transferred into 6-well cell plate and incubated at 37 °C in 5% CO_2_. The transfection efficiency was evaluated by the fluorescent counts 24 h after electroporation. Cells culture medium was half replaced by fresh complete medium containing IL-2 (100 ~ 300 U/ml) every 2 ~ 3 d. Cell viability was evaluated by flow cytometry 24 h after electroporation.

### Flow cytometry

FACSAriar (BD Bioscience) was used to perform fluorescent expression analysis. Cells were harvested the following days after transfection and stained with mouse anti-human antibody labeled by fluorescence for 30 m in 4 °C in darks as follows: CD3-PerCP-CY5.5(OKT-3, eBioscience) or CD3-FITC (HIT3a, BD Bioscience), CD4-APC (RPA-T4, BD Bioscience), CD8-PE (HIT8a, BD Bioscience), PD-1-PerCP-CY5.5 (EH12.1, BD Bioscience), CD25-PE (BC96, eBioscience), CD62L-FITC (DREG-56,e Bioscience), CD27-PE (0323, eBioscience), CD28-PE (CD28.2, eBioscience), CD45RO-PE (UCHL1, BD Bioscience), CD69-PE (FN50, BD Biolegend), HLA-DR-PE (G46-6, BD Bioscience).

### T7EN1 cleavage assay and sequencing

Cells were harvested and digested with 100 μg/ml Proteinase K in lysis buffer (10 μM Tris-HCl, 0.4 M NaCl, 2 μM EDTA and 1% SDS). Genomic DNA was extracted by phenol-chloroform and alcohol precipitation. The T7EN cleavage assay was performed as follows: briefly, targeted regions of PD1 were PCR-amplified from genomic DNA using rTaq (Takara, DR001BM) and the products were purified with a PCR cleanup kit (Axygen, APPCR-50). Purified PCR product was denatured and re-annealed in NEBuffer 2 (NEB) using a thermocycler (ABI, Veriti9902). Hybridized PCR products were digested with T7EN1 (NEB, M0302L) for 30 m and separated by 2% agarose gel. Primers for PCR are listed in Supplementary Table 3. The purified PCR products were ligated with pMD19T vector (Takara, 6013) using DNA ligation Kit Ver. 2.1 (Takara, 6022). Ligation products were used for transformation and about 20 ~ 30 colonies per kind are sequenced by using universal primer M13F.

### *In vitro* generation of autologous DC

DCs were generated from monocytes enriched by adherence for 1 ~ 2 h, and cultured in AIM-V medium containing 10% FBS together with human GM-CSF (500 U/ml, Peprotech) and IL-4 (500 U/ml, Peprotech) to obtain immature DCs. To obtain mature DCs (mDCs), fresh complete medium containing TNF-α (500 U/ml, Peprotech), IFN-α (500 U/ml, Peprotech) and PGE2 (50 ng/ml, Peprotech) was added to the culture on day 5. The culture was continued for an additional 2 d. On day 7, all DCs were harvested to be frozen or used for experiments. As described previously, these DCs possess the ability to present peptide antigen and express CD80, CD86, HLA-DR and CD11c.

### Peptides

The following peptides used for the stimulation of T cells were chemically synthesized at China Peptides (Shanghai, China): HLA-A02 restricted LMP2a 356-364 (FLYALALLL), LMP2a 426-434 (CLGGLLTMV), CEA571 (YLSGANLNL), CEA691 (IMIGHLVGV), Survivin96 (LMLGEFLKL), MUC-1 (LLLLTVLTV), and CA125 (YTLDrDSLYV). HLA-A24 restricted LMP2a 419-157 (TYGPVFMCL), MAGE-A1 (NYKHCFPEI), MAGE-A2 (EYLQLVFGI), MAGE-A3 (IMPKAGLLI), gp100 (VWKTWGQYW), tyrosinase (AFLPWHRLF) and HLA non-restricted GV1001(EARPALLTSRLRFIPK). They all achieved over 98% of purity.

### *In vitro* expansion of PD-1 KO T cells

Control T cells or PD-1 KO T-cells post transfection were cultured in AIM-V medium supplemented with IL-2 (300 U/ml, Peprotech) and half replaced by fresh complete medium containing IL-2 every 2 ~ 3 d until analyzes. For antigen stimulated T cells. Mature DCs were pulsed by peptide (25 μg/mL) for 2 ~ 3 h at 37°C, washed with pre-warmed PBS and then incubated with control T cells or PD-1 KO T-cells at a ratio of 1:10 in complete AIM-V medium supplemented with IL-2 (100 U/ml, Peprotech) in 6-well-plates (5 × 10^6 ^cells/well) on day 7 post electroporation. IL-7 and IL-15 (5 ng/mL, Peprotech) were added with fresh medium. For re-stimulation, autologous DCs were pulsed with peptide (25 μg/mL) for 2 h and added to the cultured cells for another 7 d. Fresh complete medium was added containing cytokines every 2 to 3 d until use for experiments.

### ELISPOT assay

IFN-γ ELISPOT kit (Dakewei, China) was used to determine the frequency of cytokine-expressing T cells after overnight activation with peptides. Briefly, T cells (10^5^ per well) and peptides (50 μg/ml) were added to duplicate wells and DCs were added at ratio (DC:T) of 1:5 ~ 1:10 for 18 ~ 20 h. The plates were washed before the addition of the diluted detection antibody (1:100 dilution) and then incubated for 1 h in 37 °C. After washing the plates, streptavidin-AP (1:100 dilution) was added and incubated at 37 °C for another 1 h. AEC solution mix was then added to each well, and the plates were left in the dark for about 15 ~ 25 m at room temperature before deionized water was added to stop development. Plates were scanned by Elispot CTL Reader (Cell Technology Inc, Columbia, MD) and the results were analyzed with Elispot software (AID, Strassberg, Germany).

### Cytotoxicity assay

Transduced T cells were tested for lytic activities by CFSE/PI labeling cytotoxicity assay. Target tumor cells were labeled with 4 μM CFSE (Carboxyfluorescein succinimidyl ester) (Invitrogen) for 10 m at 37 °C in PBS. Labeling was stopped by adding 10 fold volume of PBS and extensively washed in PBS before seeding into the 24-well plates. CFSE-labeled cells were then incubated with T cells by different effector to target ratio for 6 ~ 16 h. Propidium iodide (PI) (Sigma) was added to determine the ratio of cell death. Samples were analyzed by flow cytometry.

### Statistical analysis

Graphpad Prism 5.0 (Graphpad software, San Diego, CA) was used for all statistical analysis. The mean ± S.E.M. was determined for each treatment group in the individual experiments. And the one-tailed Student t-test was used to determine the significances between treatment and control group. *P-values* <0.05 were significant.

## Additional Information

**How to cite this article**: Su, S. *et al*. CRISPR-Cas9 mediated efficient PD-1 disruption on human primary T cells from cancer patients. *Sci. Rep.* **6**, 20070; doi: 10.1038/srep20070 (2016).

## Supplementary Material

Supplementary Information

## Figures and Tables

**Table 1:** The cell viability and subpopulations of *in vitro* cultured T cells.

**Donors**	**Cell viability(%)**	**CD3**^**+**^**cells(%)**	**CD4**^**+**^**cells(%)**	**CD8**^**+**^**cells(%)**
Donor1	89	96	7	91
Donor2	92	88	19	75
Donor3	94	85	13	82
Mean	92	90	13	82
SD	2	6	6	8

The percentage of viable cells 72 h after transfection and the percentage of CD3^+^ T cells, CD3^+^CD4^+^ T cells and CD3^+^CD8^+^ T cells after transfection were listed.
