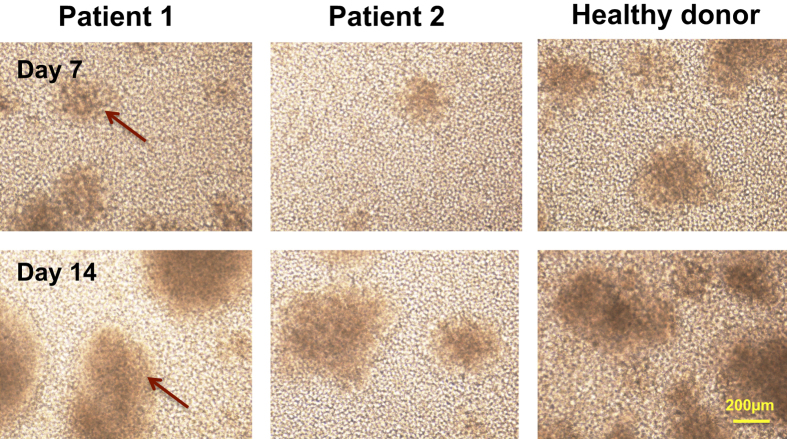# Corrigendum: CRISPR-Cas9 mediated efficient PD-1 disruption on human primary T cells from cancer patients

**DOI:** 10.1038/srep40272

**Published:** 2017-01-19

**Authors:** Shu Su, Bian Hu, Jie Shao, Bin Shen, Juan Du, Yinan Du, Jiankui Zhou, Lixia Yu, Lianru Zhang, Fangjun Chen, Huizi Sha, Lei Cheng, Fanyan Meng, Zhengyun Zou, Xingxu Huang, Baorui Liu

Scientific Reports 6: Article number: 20070; 10.1038/srep20070 published online: 01 28 2016; updated: 01 19 2017.

In figure 4d the micrograph for Patient 1 at 7 days was inadvertently duplicated for the Healthy Donor at day 7. The correct figure 4d appears below as Figure 1.

## Figures and Tables

**Figure 1:**