# Supplementary material for: CRISPR-Cas9 mediated efficient PD-1 disruption on human primary T cells from cancer patients
Source: Sci Rep. 2016 Jan 28;6:20070. doi: 10.1038/srep20070 (PMC4730182; doi:10.1038/srep20070)
Supplement: Supplementary Information [file srep20070-s1.pdf]

CRISPR-Cas9 mediated efficient PD-1 disruption on human primary T cells from cancer patients

Shu Su[1,5], Bian Hu[2,5], Jie Shao[1], Bin Shen[3], Juan Du[1], Yinan Du[2], Jiankui Zhou[2], Lixia Yu[1], Lianru Zhang[1], Fangjun Chen[1], Huizi Sha[1], Lei Cheng[1], Fanyan Meng[1], Zhengyun Zou[1], Xingxu Huang[2,4*], Baorui Liu[1*]

[1]The Comprehensive Cancer Centre of Drum Tower Hospital, Medical School of Nanjing University & Clinical Cancer Institute of Nanjing University, Nanjing 210008, China

[2]MOE Key Laboratory of Model Animal for Disease Study, Model Animal Research Center of Nanjing University, National Resource Center for Mutant Mice, Nanjing 210061, China

[3]State Key Laboratory of Reproductive Medicine, Department of Histology and Embryology, Nanjing Medical University, Nanjing 210029, China

[4]School of Life Science and Technology, ShanghaiTech University, 100 Haike Rd., Pudong New Area, Shanghai 201210, China

[5]These authors contributed equally to this work.

*Correspondence: baoruiliu@nju.edu.cn; huangxx@shanghaitech.edu.cn

Supplementary Table 1 Oligonucleotides for generating sgRNA expression vectors

Primers No.	Primer Sequences (5' to 3')
hPD1sg1up	ACCGCGTGACTTCCACATGAGCG
hPD1sg1down	AAACCGCTCATGTGGAAGTCACG
hPD1sg2for	ACCGCAGTTGTGTGACACGGAAG
hPD1sg2down	AAACCTTCCGTGTCACACAACTG
hPD1sg3for	ACCGACAGCGGCACCTACCTCTG
hPD1sg3down	AAACCAGAGGTAGGTGCCGCTGT
hPD1sg4for	ACCGGGCCCTGACCACGCTCATG
hPD1sg4down	AAACCATGAGCGTGGTCAGGGCC

Supplementary Table 2 Sequences of primers for dual sgRNA plasmids produce

Primers No.	Primer Sequences (5' to 3')
hPD1-Psg1	ATGCGTCTCAACCGCGTGACTTCCACATGAGCGGTTTTAGAGCTAGAAATAGCAAG
hPD1-Psg2	ATGCGTCTCGAAACCTTCCGTGTCACACAACTGCGGTGTTTCGTCCTTTCCACAAG
hPD1-Psg3	ATGCGTCTCAACCGACAGCGGCACCTACCTCTGGTTTTAGAGCTAGAAATAGCAAG
hPD1-Psg4	ATGCGTCTCGAAACCATGAGCGTGGTCAGGGCCCGGTGTTTCGTCCTTTCCACAAG

Supplementary Table 3 Sequences of primers for PCR amplification of targeting sites

Primers No.	Primer Sequences (5' to 3')	Amplicon (bp)
hPD1testF	GGACAACGCCACCTTCACCTGC	390
hPD1testR	CTACGACCCTGGAGCTCCTGAT	

Supplementle Figure 1 Sequences of modified hPD-1 loci detected in the HeLa cellssequencing

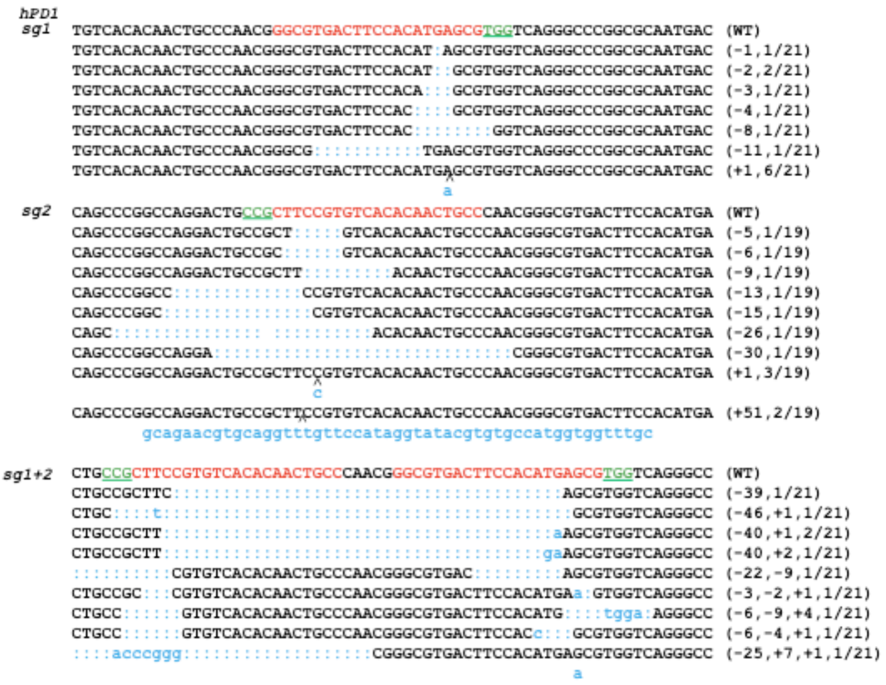

The PAM sequences are underlined and highlighted in green; the targeting sequences in red; the mutations in blue, lower case; deletions (-), insertions (+). N/N indicates positive colonies out of total sequenced.

Supplementle Figure 2 The enhanced IFN-gamma production by the Cas9-mediated disruption of PD-1 in primary T cells of cancer patients stimulated by tumor associated antigens

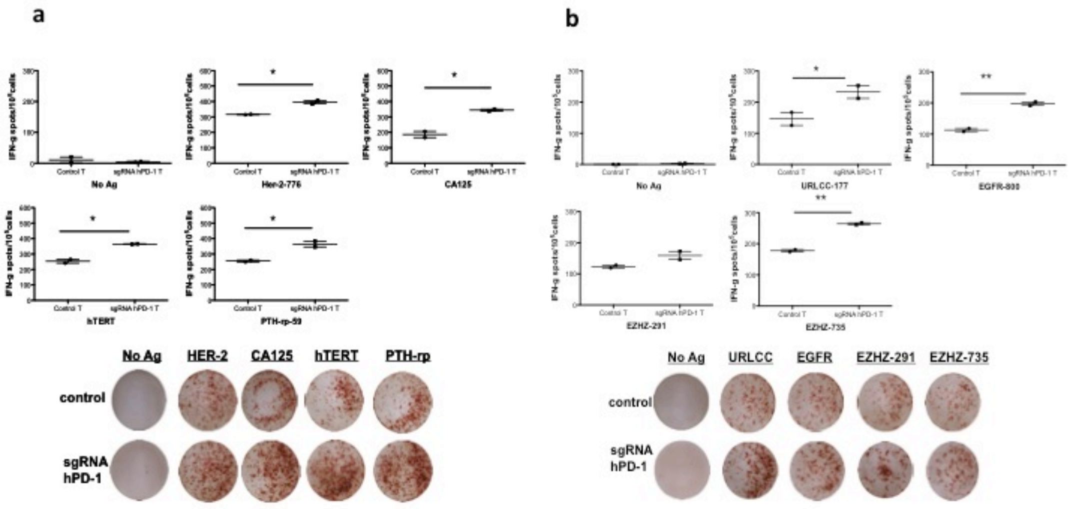

sgRNA hPD-1:Cas9 modified primary T cells and control T cells from cancer patients were cultured in IL-2 after electroporation for 7 d and stimulated by peptides pulsed autologous DCs for 20 h. The IFN-γ secreting cells was evaluated by Elispot. All values shown are mean ± SD of triplicate or duplicate measurements and have been repeated 3 times with similar results. *p <0.05; **p <0.005.
